# The Effect of Locally Administered Pamidronate on Autogenous Bone Graft in Maxillofacial Reconstruction: A Randomized Clinical Trial

**Published:** 2017-02-01

**Authors:** M. Bayat, A. Garajei, E. Afshari Pour, M. Hasheminasab, Y. Ghorbani, M. H. Kalantar Motamedi, N. Bahrami

**Affiliations:** 1 *Craniomaxillofacial Research center, Tehran University of Medical Sciences, Tehran, Iran*; 2 *Oral and Maxillofacial Surgery Department, School of Dentistry, Tehran University of Medical Sciences, Tehran, Iran*; 3 *Department of Physiology, Islamic Azad University, Tehran, Iran*; 4 *Oral and Maxillofacial Surgery, Trauma Research Center, Baqiyatallah University of Medical Sciences, Tehran, Iran*; 5 *Attending Faculty, Department of Oral and Maxillofacial Surgery, Azad University of Medical Sciences, Dental College, Tehran, Iran*; 6 *Tissue Bank and Research Center, Tehran University of Medical Sciences, Tehran, Iran*

**Keywords:** Bone transplantation, Pamidronate [supplementary concept], Reconstructive surgical procedures, Oral surgical procedures

## Abstract

**Background:**

Although bone grafts are commonly used in reconstructive surgeries, they are sensitive to local perfusion and are thus prone to severe resorption. Biphosphonates can inactivate osteoclasts and can be used to control the undesirable bone resorption.

**Objective:**

To assess the effect of administration of biphosphonates on bone resorption.

**Methods:**

20 patients with bony defects who were candidates for free autogenous grafts were randomized into “pamidronate” and “control” groups. Bone segments were soaked in either pamidronate solution or normal saline and were inserted into the area of the surgery. Bone densities were measured post-surgery and in 6-month follow-up. Data were obtained via Digora software and analyzed.

**Results:**

The mean±SD bone density in pamidronate group changed from 93.4±14.6 to 93.6±17.5 (p<0.05); in the control group the density decreased from 89.7±13.2 to 78.9±11.4 (p<0.05). The mean difference of bone density in anterior areas of the jaws showed higher DXA in comparison to posterior regions (p=0.002).

**Conclusion:**

Locally administered pamidronate affects reduction in bone resorption.

## INTRODUCTION

In reconstruction of bony defects autogenous, allogenous, or heterogenous grafts are used; autogenous bone grafts are more common. These grafts are unfortunately sensitive to local perfusion and usually undergo severe resorption. Researchers have demonstrated different solutions for this problem in years including multiple free or vascular flaps [1, 2] and synthetic materials such as demineralized freeze-dried bone (DFDB), osteoprogenitor agents like bone morphogenetic proteins (BMP) [3, 4], or membranes. Nevertheless, there is still noticeable bone resorption. Bone grafts play a significant role in improving esthetic or functional results of reconstructive surgery in maxillofacial region. The number of patients with partial or complete jaw defects is noticeable. In addition, almost all patients desire a more acceptable face as well as better functional abilities. Therefore, enhancement in bone grafting techniques for implant insertion can be of great value for these patients. After being inserted, autografts start remodeling. This process involves a catabolic phase in which the resorption of the non-vital bone graft occurs by osteoclast’s activity and an anabolic phase during which osteoblasts form a new trabecular structure [5]. It has been shown that the remodeling of bone grafts in revision surgery can be enhanced by an anabolic substance such as BMP [4]. Bisphosphonates inactivate osteoclasts and can be used to control bone resorption [3]. Bisphosphonates have strong affinity for calcium phosphate and are chemically bonded to the bone mineral [6] regardless of whether they are administered systemically [7] or locally [8]. Therefore a graft can be treated locally by simply being soaked in a bisphosphonate solution before implantation, hence, protecting the new-forming bone from resorption [4]. This study was designed to determine if locally administrated bisphosphonates can affect bone graft resorption [9].

## MATERIALS AND METHODS

Twenty patients with bony defects who were candidates for free autogenous bone graft in jaws were included in this study. The study protocol was approved by Ethics Committee of Tehran University of Medical Sciences (code 86-02-70-5483). Exclusion criteria consisted of any systemic disease that can disturb bone formation or resorption.

Patients were randomized into study and control groups. Each patient received 1 g cefazolin IV, prophylactically at the beginning of the surgery. At first, the intra-oral recipient site was prepared. Then, corticocancellous block was harvested from the anterior part of iliac bone. The average size of grafts was 4 cm. In the study group, bone blocks were soaked in pamidronate solution (10 mg/mL) for 10 min. After graft implantation and fixation with plate and screws (synthesis company), a water-tight closure was achieved with vicryl 4-0 suture. Patients stayed in hospital for three days. During hospitalization 1 g cefazolin IV, was administered every six hours during the first 10 days post-operative. In six-month follow-up visit, panoramic radiography was taken (Cranex D digital panoramic cephalometric x-ray unit, current: 10 mA, voltage: 73 kV, time: 17.6 sec).

**Figure 1:**
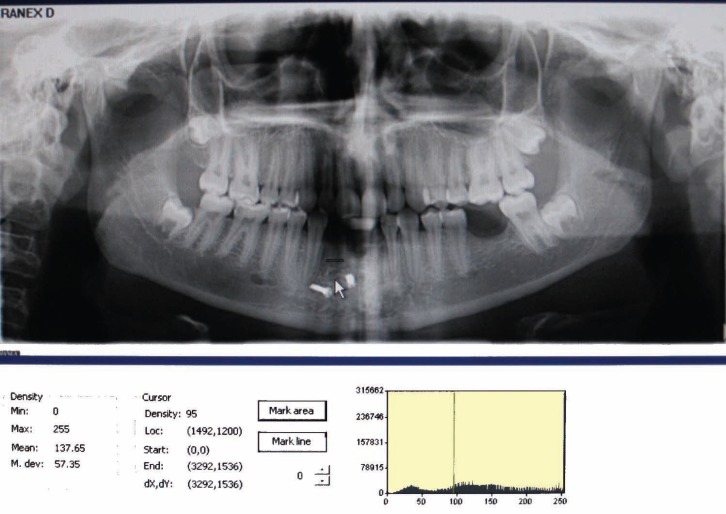
An screen shot of Digora software

Bone density was measured with Digora^®^ for Windows^®^ (DFW) (Soredex company) ver 2.7 (Fig. 1). To measure bone density in one region, five sites from alveolar crest to closest anatomic landmark parallel to the sagittal plane were chosen and bone density in each site was measured. The mean value of these five readings was considered the total bone density in that region. 

Statistical Analysis

Statistical analyses were performed with SPSS^®^ for Windows^®^ ver 18.0 (Statistical Product and Service Solutions, SSPS Inc., Chicago, IL, USA). Results are presented as mean±SD. *Student’s t *test was used to compare means of two normally distributed continuous variables. A p value <0.05 was considered statistically significant. All tests were two-sided.

## RESULTS

**Figure 2:**
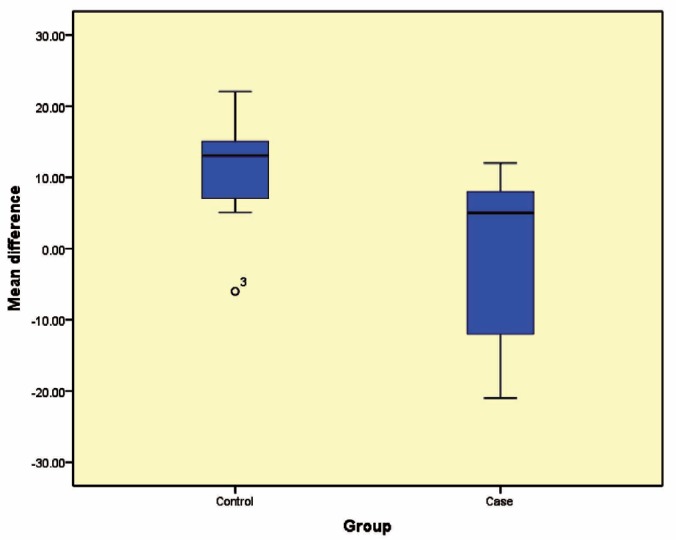
Mean difference in two studied groups

**Figure 3:**
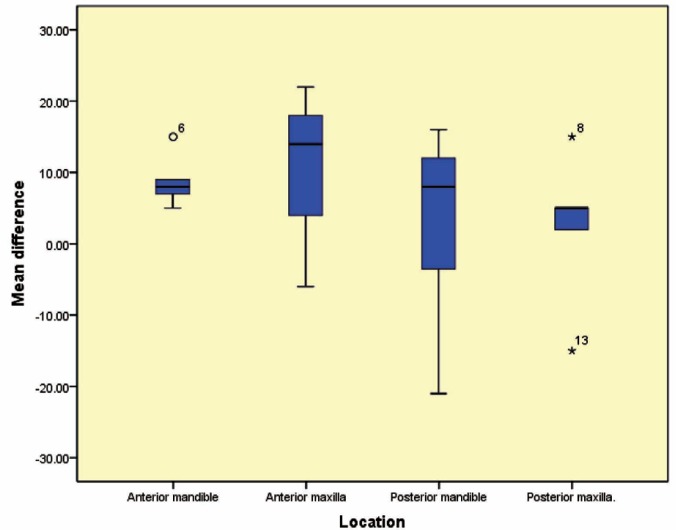
Mean difference in various locations

Twenty patients were evaluated out of whom, 10 were allocated to pamidronate group and 10 served as the control group. The mean±SD bone density measured by dual-energy X-ray absorptiometry (DXA) in the control group decreased from 89.7±13.2 to 78.9±11.4 (Table 1); in the study group, it increased from 93.4±14.6 to 93.6±17.5 (Table 2, Fig. 2). The bone density in the anterior maxilla was 10.0±14.4; in the anterior mandible, it was 8.8±3.7. Although these changes in comparison to the posterior maxilla (2.4±10.8) and posterior mandible (2.8±13.9) were higher, they were not statistically significant (p=0.665) (Table 3, Fig. 3).

**Table 1:** Bone densitometry in the control group

Patient	DXA before	DXA after	Mean difference
1	95.0	73.0	22.0
2	84.0	70.0	14.0
3	71.0	77.0	-6.0
4	83.0	76.0	7.0
5	114.0	98.0	16.0
6	94.0	79.0	15.0
7	83.0	78.0	5.0
8	80.0	65.0	15.0
9	108.0	100.0	8.0
10	85.0	73.0	12.0

**Table 2:** Bone densitometry in the study group

Patient	DXA before	DXA after	Mean difference
11	94.0	89.0	5.0
12	98.0	93.0	5.0
13	76.0	91.0	-15.0
14	94.0	85.0	9.0
15	85.0	73.0	12.0
16	80.0	72.0	8.0
17	111.0	123.0	-12.0
18	84.0	105.0	-21.0
19	87.0	85.0	2.0
20	125.0	120.0	5.0

**Table 3:** Comparison in two Groups. Values are mean±SD.

	Control	Case	Total
DXA after	78.9±11.4	93.6±17.5	86.3±16.2
DXA before	89.7±13.2	93.4±15.0	91.6±13.8
Mean difference	10.8±7.7	-0.2±11.4	5.3±11.1

## DISCUSSION

One of the most common problems of autogenous graft in reconstructive surgeries in maxillomandibular region is bone resorption. This study was focused on catabolic activity of osteoclasts to reduce bone resorption. A similar study on the effect of alendronate application on transplanted bone revealed that bone density in the study group after six months was higher than the control group [9]. This effect can be attributed to anti-catabolic effect of bisphosphonate, which can lead to both higher bone volume and density. Our results were comparable to some previous reports, which show preventive effects of biphosphonates on bone resorption [3, 8, 10]. Data analysis showed that the mean difference in posterior region of jaws (max: 2.4±10.8, mean: 2.8±13.9) was less than the anterior region (max: 10.0±14.4, mean: 8.8±3.7). In some cases, DXA was even increased following pamidronate application—mostly in bone grafts that were placed in the posterior region. This can be interpreted by taking into account that functional stress is higher in the posterior region. It was also shown that in cases treated with bisphosphonates, bone graft was replaced with new bone and bone resorption was reduced, so that the new bone formed was a combination of old and new structures [4]. In our study bone density was reduced in both groups, but this reduction in the control group (89.7 to 78.9) was more than that observed in the study group (91.6 to 86.3).

In a study conducted in 2003 on a group of rats, Kaynak, *et. al.* [3], systemic alendronate was administered and its effect on bone resorption after elevation of mucoperiosteal flaps, was studied. They reported that bone resorption in the study group was significantly lesser than that in the control group. Aspenberg also demonstrated that alendronate can reduce autograft resorption [8]. In a study conducted in 2006, Kesteris, *et. al.* [10], showed that soaking bone grafts in bisphosphonate solution can effectively reduce their resorption.

The effect of bisphosphonates on graft resorption was measured by evaluating BMP, ALP, and mRNA expression, which are correlated with bone resorption. It was concluded that bisphosphonates can decrease expression of genes that are related to bone resorption [11]. Atrand, *et. al.* [12], found that systemic administration of zolendronic acid, can reduce bone resorption in both newly formed bone and bone grafts.

Gursoy and Altundal, *et. al.* [13, 14], reported that alendronate can enhance new bone formation and reduce bone resorption after tooth extraction. Gupta, *et. al.* [15], showed that use of alendronate can improve bone formation in infrabony defects.

The effect of alendronate supplemented with cefazolin on allografts was evaluated in a case-control study in goats. The study showed application of alendronate and cefazolin reduces bone resorption [16]. Systemic and local administration of alendronate showed identical results in new bone formation in distraction osteogenesis. Bone healing was also significantly improved in those who received either systemic or local alendronate [17].

A study conducted in 2007 [18], showed that soaking morselized allograft in alendronate solution can impair implant fixation. According to Belfrag [4], several advantages can be obtained by local application (instead of systemic administration) of bisphosphonate. With local application, the drug is added to the graft already during the operation. All of the graft will be treated independently of the level of revascularization, and all bone binding sites will be saturated with chemically bound pamidronate, thus protecting the graft from resorption until completely remodeled with no concern regarding the drug being washed out. Furthermore, by local administration of the drug, poor patient compliance is avoided. Moreover, the systemic effects of an intravenous bisphosphonate can be avoided and the total dose of the drug used is less than systemic administration. Guniz Akdenic conducted a study to evaluate bone density in peri-implant bone by panoramic radiographs [19]. 

To figure out if locally administered bisphosphonate remains local or is washed out and absorbed systemically, McKenzie, *et. al.* [20], conducted a study and showed that local bisphosphonate remained around porous implants and no effect was observed on other bones. Therefore, local bisphosphonate solution reduces the risk of systemic side effects and skeletal bisphosphonate exposure. Further studies are needed to further study the routine administration of local pamidronate in grafts, especially with respect to graft’s mechanical strength.

## Conflicts of Interest:

None declared.

## FINANCIAL SUPPORT:

This study was supported by a grant (#90-01-106-11742) from Craniomaxillofacial Research Center, Tehran University of Medical Sciences, Tehran, Iran.
